# Dietary Exposure to Particles of Polytetrafluoroethylene (PTFE) and Polymethylmethacrylate (PMMA) Induces Different Responses in Periwinkles *Littorina brevicula*

**DOI:** 10.3390/ijms24098243

**Published:** 2023-05-04

**Authors:** Victor Pavlovich Chelomin, Valentina Vladimirovna Slobodskova, Sergey Petrovich Kukla, Andrey Alexandrovich Mazur, Nadezda Vladimirovna Dovzhenko, Avianna Fayazovna Zhukovskaya, Alexander Alexandrovich Karpenko, Maxim Alexandrovich Karpenko, Vyacheslav Sergeevich Odintsov

**Affiliations:** 1 Il’ichev Pacific Oceanological Institute, Far Eastern Branch, Russian Academy of Sciences, 690041 Vladivostok, Russiakukla.sp@mail.ru (S.P.K.);; 2 A.V. Zhirmunsky National Scientific Center of Marine Biology, Far Eastern Branch, Russian Academy of Sciences, 690041 Vladivostok, Russia

**Keywords:** microplastics, gastropods, genotoxicity, oxidative stress

## Abstract

The marine and ocean water pollution with different-sized plastic waste poses a real threat to the lives of the next generations. Plastic, including microplastics, is found in all types of water bodies and in the organisms that live in them. However, given the chemical diversity of plastic particles, data on their toxicity are currently incomplete. Moreover, it is clear that different organisms, depending on their habitat and feeding habits, are at different risks from plastic particles. Therefore, we performed a series of experiments on feeding the gastropod scraping mollusk *Littorina brevicula* with two types of polymeric particles—polymethylmethacrylate (PMMA) and polytetrafluoroethylene (PTFE)—using a special feeding design. In the PMMA-exposed group, changes in gastrointestinal biochemical parameters such as increases in malondialdehyde (MDA) and protein carbonyls (PC) were detected, indicating the initiation of oxidative stress. Similarly, a comet assay showed an almost twofold increase in DNA damage in digestive gland cells compared to the control group. In mollusks fed with PTFE-containing food, no similar changes were recorded.

## 1. Introduction

In recent years, ecotoxicological research has been strongly dominated by the spread of synthetic polymers, primarily “microplastics” (MP), into the biosphere [1,2]. Analysis of scientific data shows that the main efforts of numerous research teams have focused on monitoring MP in various environments, identifying regions of high MP concentration, changes over time, and assessing the risk of MP transmission through food chains. As a result, it has become apparent that the distribution of MP is global and MP particles have been found in all environments (water, land, air, and biota) [2,3,4,5,6,7]. In particular, in the global ocean, MP fragments are even present in the waters of the Arctic and Southern Oceans and at the bottom of deep-sea trenches [3,4]. Moreover, MP presence has been detected within organisms of different systematic and trophic levels [5,6,7,8,9,10]. Assessing the extent of spread and penetration of synthetic polymer particles into the biosphere, it is logical to assume that MP represents a potential ecological threat, as it can start the initiation of ecosystem transformation processes.

As a result, studies aimed at identifying the toxic characteristics of synthetic polymers are of particular significance. The number of publications evaluating the potential effects of ingestion of various plastic fragments by different organisms is steadily increasing [11,12,13,14,15]. To date, several key mechanisms of MP interactions with classical ecotoxicological models such as bivalves, crustaceans, and fish have already been investigated [16,17,18,19,20]. However, to understand the real situation in the marine environment, it is necessary to study these mechanisms in different taxonomic groups of marine fauna with specific feeding behavior.

In this fact, the group of littoral gastropods is of particular interest, typical representatives of which are species of the family *Littorina*. These organisms are at increased risk of interacting with artificial polymers, as they inhabit predominantly the littoral zone, where the main mechanical breakdown of large plastic fragments to MP occurs [21]. In addition, individuals of this family are characterized by a particular feeding strategy of scraping food particles from the surface of phytobenthos, which also contributes to the interaction and ingestion of different-sized plastic particles [21,22,23]. At the same time, according to experimental data, mollusks do not distinguish between food and polymeric particles [22].

It has been suggested that these gastropods may be more susceptible to the potentially damaging effects of MP, as they absorb more of them per body mass than other littoral species [21]. While the mechanisms of uptake, distribution in the digestive system, and excretion of MP particles in these mollusks are, at present, fairly well understood, the effects on physiological and biochemical processes have not been sufficiently addressed.

Thus, we made a series of experiments on the far-eastern gastropod *Littorina brevicula* to evaluate the effects of different-sized microparticles of plastic polymethylmethacrylate (PMMA) and polytetrafluoroethylene (PTFE) in contact with cells of the digestive system on biochemical mechanisms involved in the formation of oxidative stress and genotoxicity. For this purpose, we used the original food model proposed earlier [24], allowing not only control of littoral feeding conditions, concentration, and absorption of MP particles, regardless of their buoyancy, but also a collection of fecal residues and monitoring of physicochemical changes on polymer surfaces during transit through the mollusk digestive system.

A mixture of PTFE and PMMA microparticles widely used in human household and economic sectors were used as model particles to study the biological effects of plastics [25,26,27]. According to current polymer classifications [28,29], which are based on different criteria, PMMA is categorized as a potentially hazardous polymer, whereas PTFE, due to lack of sufficient information, is not classified.

Toxicological studies involving different systematic groups with different feeding behavior contribute to a better understanding of the key mechanisms of interaction between synthetic polymers and biochemical systems and may also be useful for improving the predictive assessment of the environmental and human health impacts of plastic pollution.

## 2. Results and Discussion

Plant-eating gastropods, a typical example of which is *Littorina brevicula*, feed by scraping phytosubstrate with a special organ called a radula [30]. In search of food particles, these mollusks excrete mucus, which has sticky properties, onto the surface of algae [22,31]. The deposited food particles and fragments of MP are retained by the mucus and, subsequently, carefully scraped off using the radula. In this respect, the method of feeding *L. brevicula* with a food substrate containing fragments of different-sized particles of two types of plastic (PMMA, PTFE) proposed earlier [24] and applied in our work, to a certain extent, imitates the natural way of feeding typical for these mollusks.

A serious problem remains the lack of information on the concentrations of plastic particles on the surface of phytobenthos. Accordingly, the concentration of polymer particles in the food substrate used in our experiments (4 mg/cm^2^) is difficult to compare with the real ones in the environment. Therefore, in our study, we did not limit ourselves to determining the minimum effective concentrations based on the dose–response relationship but rather to identifying the sensitivity of the organism’s biochemical systems to degraded polymer particles during their passage through the digestive tract.

According to experimental data, gastropods do not differentiate between food and polymeric particles; thus, the MPs uptake with food enters the digestive system and, after some time, comes out with fecal pellets [22,23]. A similar pattern with the same plastic particles was observed in the experiment of Odintsov et al.: within a few hours, fragments of both types of plastic were recorded in the pellets. To control the physicochemical changes on the polymer surface during passage through the mollusk digestive system [24], fecal particles with MPs were again added to food agar plates for feeding (the cycle was repeated six times). During the experiments, no visible changes in feeding behavior and activities were recorded in the experimental mollusks compared to the control groups.

**Figure 1:**
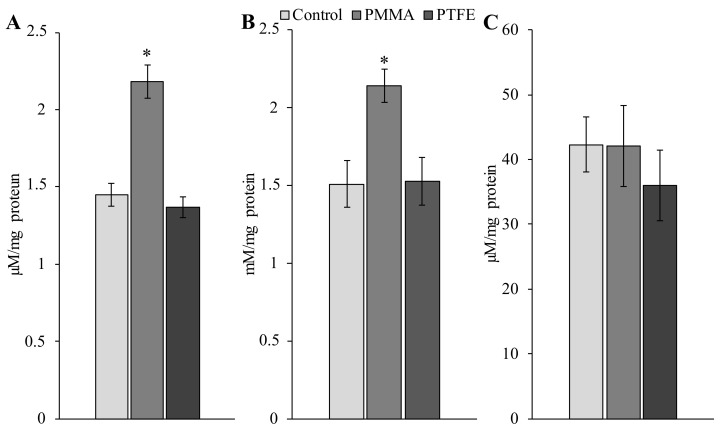
Effect of different-sized polymethylmethacrylate (PMMA) and polytetrafluoroethylene (PTFE) particles on the levels of MDA (**A**), PC (**B**), and IAA (**C**) in cells of the digestive gland of *L. brevicula* (mean ± standard deviation, *n* = 27); *—difference from control was significant at *p* ≤ 0.05.

According to the biochemical results, the experiments with the diet containing MPs of both types of polymers showed that only the PMMA-induced biochemical shifts in the mollusk digestive cells were typical for the development of the oxidative stress processes. This was evidenced by reliable accumulation products of lipid peroxidation (LPO): levels of malonaldehyde (MDA) and protein carbonyls (PC) (Figure 1A,B, respectively).

According to the literature data, the obtained results are typical not only for the interaction of this type of plastic with *L. brevicula*. In this context, it is well known that different types of polymers, when interacting with marine organisms of different trophic levels and feeding types, usually have no noticeable effect on survival. Nevertheless, MPs caused various sublethal effects at a molecular and biochemical level related to the generation of reactive oxygen species (ROS) and the development of oxidative stress processes [11,32,33,34,35,36,37,38]. Although the detailed mechanism of ROS generation upon exposure of chemically inert polymer particles to biological systems is still unclear, the accumulation of lipid and protein peroxidation products (MDA and PC, respectively) are generally recognized as early and sensitive indicators of increased ROS generation and oxidative stress [11,39]. It should be particularly noted that we did not observe reliable changes in the level of antiradical potential (IAA-TOSC) of digestive cells in both experimental groups of litorin against the background of the accumulation of these products’ degradation of lipids and proteins (Figure 1C). A similar pattern occurred in the work of Avio and colleagues [32], in which MPs caused a decrease in lysosomal membrane stability (LSM) as a result of induction of ROS generation, with little or no effect on TOSC levels. Comparing these above data, it is logical to assume that the marker of oxidative stress IAA-TOSC, in this case, is less sensitive than the molecular biomarkers MDA, PC, and LSM or can be referred to as the markers that change under longer exposures.

**Figure 2:**
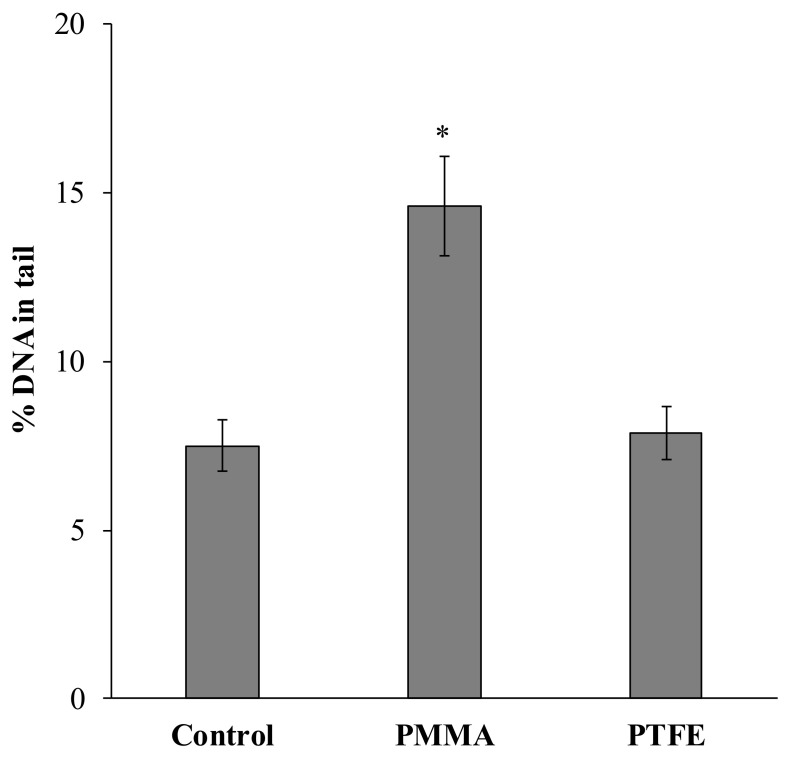
Level of nuclear DNA damage in *L. brevicula* digestive cells after feeding food containing PMMA and PTFE microparticles (mean ± standard deviation, *n* = 27); *—difference from control was significant at *p* ≤ 0.05.

In addition to the induction of peroxidation of basic biomolecules (lipids and proteins), exposure of *L. brevicula* to PMMA-containing food led to a 2-fold increase in the ratio of fragmented nuclear DNA migrating, according to the DNA comet method, from the nucleus to the “tail” of the comet (Figure 2).

**Figure 3:**
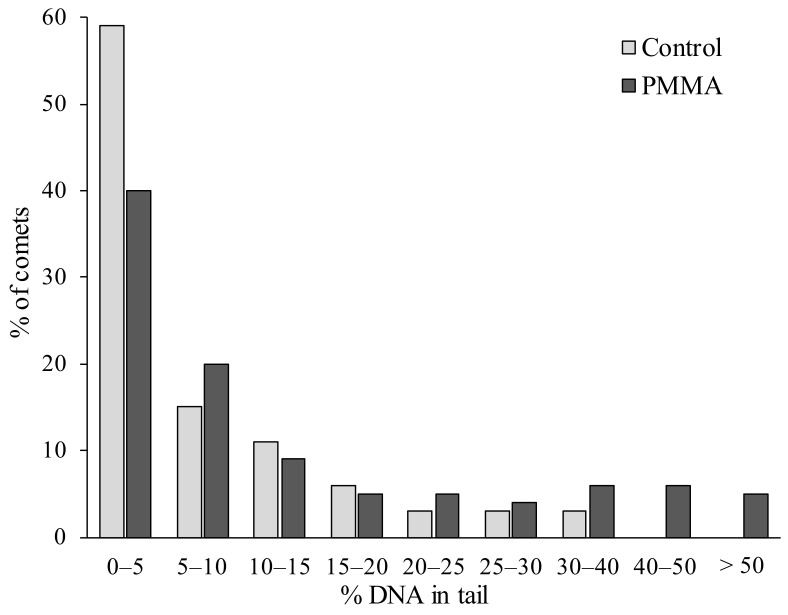
Distribution of comets according to the degree of nuclear DNA damage in *L. brevicula* fed with Protacryl m particles.

For a more detailed analysis, the comets that were formed from digestive cell DNA in control and experimental mollusks were grouped by the level of genome fragmentation and presented in Figure 3.

In this case, it can be seen that the level of nuclear DNA fragmentation did not exceed 20–25% in 95% of cells in the digestive gland of the control groups of the gastropods. Whereas, in the experimental mollusks after feeding with PMMA, the proportion (<80%) of such cells with a relatively low level of genome damage sharply decreased. In addition, comet cells with more than 40–50% of DNA migrating to the “tail” were recorded. Overall, these results show that PMMA microparticles, entering the digestive tract of *L. brevicula*, exhibited biological activity in the genotoxicity type. In this regard, our results, although obtained with PMMA, were consistent with several studies obtained when different types of polymers were exposed to different biological models [11,14,39,40,41,42,43]. Apparently, the biochemical processes that result in increased DNA damage in our experiments and other studies were of a universal nature and were related to ROS generation [44,45].

Despite the growing interest in the genotoxic properties of various types of MPs, the question of how chemically inert polymers, entering biological systems, induce these negative biochemical reactions remains the subject of discussions.

It should be admitted that PMMA MPs were not retained in the *L. brevicula* digestive tract and were rapidly excreted with fecal residues in our experiments. It follows from this that the distinctive feature of our results is that the development of oxidative stress processes and genome damage in mollusk digestive cells are registered, apparently, without the intracellular accumulation of polymer particles. There is reason to believe that the biochemical effects inside the cell could be induced directly by polymer particles without penetration inside the cell. In this case, MPs, absorbed on the cell membrane surface, act as a physical factor initiating the receptor-signaling mechanism of the cell, as has been the case in several studies [46,47,48]. However, given that similarly sized PTFE particles did not induce any biochemical effect (Figure 1 and Figure 2), we consider this mechanism unlikely.

The possible causes of the negative biochemical shifts shown in our experiments with PMMA may be the chemical compounds present in this polymer. It is probable that they enter during synthesis or are formed as a result of physicochemical or biological degradation processes when the particles pass through the digestive system of *L. brevicula*.

According to the classification of polymers ranked based on their chemical composition in terms of environmental hazard, PMMA is classified as a highly hazardous polymer [28,29]. It is noteworthy that PTFE is not ranked by hazard, due to a lack of experimental data on toxicity, unlike PMMA. PTFE is a soft plastic that is considered to be the most inert material known. It is resistant to gastric juice and is extremely stable. In confirmation of the inertness of this polymer, Naftalovich and colleagues [49] have experimentally shown that rats fed a diet containing 25% PTFE for 90 days showed no signs of toxicity. Furthermore, based on these results, they have suggested that this polymer could be used for food supplementation to significantly improve satiety and reduce caloric intake in humans.

The high toxic risk of PMMA is related to the presence of a series of acrylic acid derivatives in the polymer structure, which, by diffusing from the polymer, can enhance the generation of ROS and cause genotoxicity [50,51,52,53]. A good example is experiments showing that cytogenetic and genotoxic effects were detected with the exposure of sea urchin sperm (*Sphaerechinus granularis*, Paracentrotus lividus) with PMMA microparticles or with extracts of this polymer [54,55].

Based on the above studies and our results, it can be suggested that the PMMA structure contains relatively labile chemical components that induce damage to the cellular genome during passage through the digestive tract. Although specific studies of these reactive substances are beyond the scope of our work, it should be emphasized that the internal environment of the digestive system of *L. brevicula* may contribute to their mobilization from the polymer structure. In the clam digestive tract, microplastic particles are exposed to specific physicochemical conditions and may be exposed to digestive enzymes, ROS, and reactive metabolic intermediates that contribute to the chemical modification of the polymer. In addition, the presence of symbiotic microorganisms in the digestive tract of *L. brevicula* cannot be excluded, which may also contribute to the modification of the PMMA structure. These notions are based on the results of [24], in which it was convincingly shown that fragments of PMMA microparticles underwent significant physicochemical modifications after being in the digestive tract of *L. brevicula*. Such modifications in the polymer structure accompanied by changes in the physical and mechanical properties of PMMA particles may lead to a weakening of the forces holding non-covalently bound endogenous chemicals, including various synthesis additives and products of incomplete polymerization, in the polymer structure.

Biochemical system sensitivity for maintaining genome stability in *L. brevicula* to exposure to PMMA polymer microparticles, revealed in our work, is regarded as the initial stage of research, based on which we can, to a certain extent, estimate dose-dependent effects for the identification of minimum effective concentrations of plastic and how they correspond to the real ones in the marine environment.

Although a number of the statements made in our work are hypothetical, we suggest that dangerous toxic effects are not only determined by the concentration of plastic particles but are to some extent determined by the nature of the physicochemical modification of the polymer, which may significantly affect its bioavailability and toxicity mechanisms, when polymer particles penetrate the gastrointestinal tract.

## 3. Material and Methods

### 3.1. Description of the Experiment

Adult gastropods *Littorina brevicula* (mollusk shell height 10 ± 1 mm) were collected in the intertidal zone of Alekseev Bay (Peter the Great Bay, Sea of Japan). This species is a mass inhabitant of the coastal zone in the seas of the northwestern Pacific Ocean. The mollusks were divided randomly into three groups of 30 individuals each. Then, mollusks from each group were divided into 10 individuals and kept in three parallel glass tanks. All organisms were fed using the food model previously described [24]. For this purpose, plates (10 cm^2^) with 2 mL of dried seaweed (Porphyra) aqueous extract (Sin Young Food Co., Busan, Republic of Korea) were prepared based on 4% agar–agar. Similar plates were prepared for the experimental groups, but 40 mg of MPs of 0.1–10 µm polytetrafluoroethylene (PTFE) (Halopolymer Co., Ltd., Moscow, Russia) and 30–100 µm polymethylmethacrylate (Protacryl-M) (Nikadent Co., Ltd., St. Petersburg, Russia) were added, respectively. On the following day, after the food model had been consumed by animals, pellets were collected from the bottom of the cylinder and added to the next portion of food. This procedure was repeated six times. The total duration of the experiment was 7 days. Due to their physicochemical characteristics, both polymers were well-identified by microscopy and Raman microspectroscopy in both water and animal products [24].

On the seventh day, after 6 feeding cycles, the digestive gland of *L. brevicula* was used for biochemical studies. Nine specimens of mollusk were taken from each aquarium to determine the level of LPO and the degree of DNA damage. All procedures in the present work, as well as the mollusks disposal methods, were approved by the Commission on Bioethics at the V.I. Il’ichev Pacific Oceanological Institute, Far Eastern Branch of Russian Academy of Science (protocol №16 and date of approval 15 April 2021), Vladivostok, Russia.

### 3.2. Comet Assay

To determine the amount of damage in the DNA molecule, we used the alkaline version of comet analysis [56], which successfully adapted to marine organisms. This approach is a promising modern diagnostic and prognostic tool for revealing hidden pathological changes in any organism [40]. Visualization and registration of DNA comets were performed using a scanning fluorescence microscope (Zeiss, Oberkochen, Germany, AxioImager A1) equipped with an AxioCamMRc digital camera. The computer program CASP software v 1.2.2 was used to process digital images. (CASPLab, Wroclaw, Poland; https://casplab.com, accessed on 15 March 2023), which allowed the calculation of various comet parameters that indicated the degree of damage to cellular DNA. For each comet, the proportion of DNA in the comet’s tail was calculated.

### 3.3. MDA Concentration

To determine malondialdehyde and the index of antiradical activity, the digestive gland was homogenized in 0.1 M phosphate buffer pH 7.0. The content of malondialdehyde (MDA), a product of oxidative degradation of fatty acids, was determined in tissues and subcellular fractions by color reaction with thiobarbituric acid (TBA, Merck KGaA, Darmstadt, Germany. CAS-no 504-17-6) [39,57]. The measurements were carried out at a wavelength of 580 nm and 532 nm, then the difference in the readings of the optical density was found. To calculate the MDA content, the molar extinction coefficient was used—1.56 × 10^5^/cm/M. The relative content of MDA was expressed in µmol per mg of protein. The measurements were carried out on a Shimadzu UV-2550 spectrophotometer.

### 3.4. Integral Antioxidant Activity

The determination of the Integral antiradical activity of the digestive gland of mollusks was carried out by a spectrophotometric method based on the ability of the cellular antioxidant system to recover the radical cation ABTS+ (2, 2-azinobis 3-ethylbenzothiazoline 6-sulfonate), an oxidation reaction of ABTS+ by peroxyl, and alkoxyl radicals that result from thermal degradation of 2, 2-azobis (2 aminopropane) hydrochloride (ABAP). The reaction mixture was prepared in 0.1 M phosphate buffer (pH 7.0) and incubated at 37 °C [39]. The measurements were made using a Shimadzu UV-2550 spectrophotometer with a thermostatted cell at a wavelength of 414 nm. The magnitude of activity was calculated with a calibration plot using trolox (6-hydroxy-2,5,7,8-tetramethylchloraman-2- carboxylic acid, Sigma Aldrich, Taufkirchen, Germany).

### 3.5. Carbonyl Concentration

For the determination of protein carbonyls, the digestive gland was homogenized in 0.05 M phosphate buffer pH 7.0 supplemented with 1 mM PMSF (phenylmethanesulfonyl fluoride) to inhibit proteases. Carbonyl groups of proteins in the digestive gland and gills were determined by the alkaline method [39]. In total, 400 µL of DNPH (10 mM in 0.5 M H_3_PO_4_) was added to 400 µL of protein solution followed by a 10 min of incubation in dark. Then, 200 µL of NaOH (6 M) was added. Time exposure this mixture was 10 min as well. Absorbance was read at 450 nm by spectrophotometer Shimadzu UV-2550. The concentration of carbonyl groups (mMol/mg of proteins) in the investigated solutions was calculated using the molar absorptivity of 22,000 M^−1^ cm^−1^. The Lowry method was used to determine the protein concentration [58]. The Lowry method is based on two reactions. The first one consists of the formation of a complex of copper cations (Cu^2+^) with amide bonds of the protein, followed by the reduction of copper under alkaline conditions. The resulting product is called biuret chromophore, which is stabilized by the addition of tartrate. In the second reaction, the resulting copper–protein complex is reduced with Folin’s reagent. In this case, the protein solution turns blue. The transparency of the solution is determined spectrophotometrically in the wavelength at 660 nm. Calibration curves were built using solutions of bovine serum albumin, the concentrations of which were calculated based on the molar extinction coefficient.

### 3.6. Statistical Analysis

The experiment results were processed with MS Excel 2003 and Statistica 10 software package (StatSoft, Tulsa, OK, USA; http://statsoft.ru/resources/support/download.php, accessed on 2 May 2023). For the data, nonparametric Kruskal–Wallis ANOVA followed by pairwise Mann–Whitney tests were performed. A difference of *p* < 0.05 was considered statistically significant.

## 4. Conclusions

In this study, the dietary exposure of PTFE and PMMA particles to the induction of lipid and protein peroxidation and nuclear DNA damage in *L. brevicula* digestive cells was studied for the first time. Clearly, the biochemical disorders induced by PMMA polymer particles are more diverse and are not limited to the oxidative stress and genotoxicity identified in our work. However, within the considered problem, the ability of these polymer particles to induce the disruption of mollusk digestion cells’ involved genome structures is of the greatest interest. Due to the importance of genome integrity in carrying out the basic functions of any biological system, there is reason to be concerned about the genotoxic properties of PMMA with the risk of developing long-term negative consequences of this effect. Additionally, the comet assay applied in our work makes it possible to evaluate changes in the cell nucleus at the early stages of genome destruction; therefore, the identified genotoxic properties of PMMA particles are not only diagnostic but also prognostic.

## Data Availability

Not applicable.
